# Prevalence and Evolution Analysis of Porcine Circovirus 3 in China from 2018 to 2022

**DOI:** 10.3390/ani12121588

**Published:** 2022-06-20

**Authors:** Dengjin Chen, Yi Huang, Yating Guo, Lihong Wang, Yongning Zhang, Lei Zhou, Xinna Ge, Jun Han, Xin Guo, Hanchun Yang

**Affiliations:** Key Laboratory of Animal Epidemiology of the Ministry of Agriculture, College of Veterinary Medicine, China Agricultural University, Beijing 100193, China; chendengjin-cau@foxmail.com (D.C.); s20193050752@cau.edu.cn (Y.H.); guoyating-cau@foxmail.com (Y.G.); wlh-cau@foxmail.com (L.W.); zhangyongning@cau.edu.cn (Y.Z.); leosj@cau.edu.cn (L.Z.); gexn@cau.edu.cn (X.G.); hanx0158@cau.edu.cn (J.H.); yanghanchun1@cau.edu.cn (H.Y.)

**Keywords:** prevalence, phylogenetic analysis, porcine circovirus 3

## Abstract

**Simple Summary:**

An increasing number of studies have shown that the PCV3 virus causes signs and symptoms similar to PDNS in pigs, and since first being identified in the United States, it has caused reproductive failure in pigs. Studies have shown that it has spread worldwide, especially in China. However, to date, there are only a few reports of PCV3 detection and sequence variation, and limited information is known about its distribution in China’s major swine-producing regions. This study examined the prevalence of PCV3 in China and its evolutionary relationship. A high level of PCV3 infection has been found in serum samples, and it has been found that PCV3 infection has a widespread distribution among Chinese pig herds. The ORF2 genes of the strains were analyzed and compared with other PCV3 strains, which were downloaded from the NCBI. Our phylogenetic analysis indicated a close relationship with the strains previously described in pigs, and additional analysis revealed that all isolates obtained in this study could be divided into two sub-clades: 3a and 3b. Overall, this study showed that PCV3 prevalence in China is high and there is a lot of genetic divergence among the strains, which may pose a threat to the porcine industry.

**Abstract:**

Porcine circovirus 3 (PCV3) is an emerging virus, causing substantial economic losses in pig populations, that was first detected in 2016. Furthermore, the virus has already been reported in Europe, the Americas, and Asia, including China, indicating that the virus has spread worldwide. However, the molecular epidemiology of PCV3 still needs further study. To investigate PCV3 epidemiological characteristics in China, 2707 serum samples of pigs were randomly collected from 17 provinces in China between September 2018 and March 2022 and analyzed via PCR assays. The study showed that PCV3 infection was prevalent in the overall population with 31.07% (841/2707) and 100.0% (17/17) at sample and province levels, respectively, though the positivity rate of PCV3 varied from 7.41 to 70.0% in different provinces, suggesting that PCV3 infection has a widespread distribution in China. We selected 22 serum samples from different regions that had high levels of viral DNA for amplification and sequenced their ORF2 (Cap) gene. According to the phylogenetic analysis, all isolates in the current study could be grouped into two separate subclades, with 15 strains belonging to clade 3a and 7 strains belonging to clade 3b, indicating that PCV3a and PCV3b were the predominant subtypes in the regions of China studied. Meanwhile, additional analysis revealed that the capsid gene sequences identified in this study displayed 97.46~99.8% nucleotide (nt) and 97.06~100% amino acid (aa) sequence similarity with other PCV3 available reference strains, respectively. In general, our studies provide important insights for understanding the prevalence and evolution of PCV3 in China and will guide future efforts to develop measures for preventing and controlling the disease.

## 1. Introduction

Porcine circoviruses (PCVs), especially PCV2 and PCV3, are very common in pigs and can lead to diverse clinical presentations, including post-weaning multisystemic wasting syndrome (PMWS), reproductive failure, porcine dermatitis and nephropathy syndrome (PDNS) [1,2,3], and so on, which can cause significant economic losses in the global swine industry, including in China. PCVs, belonging to the genus *Circovirus* of the family *Circoviridae*, are small, non-enveloped, and single-stranded circular DNA viruses [4], and they are the smallest viruses that replicate on their own [5]. There are at least four PCV species known thus far: porcine circovirus 1 (PCV1), PCV2, PCV3, and PCV4, respectively. In 1974, PCV1 was first discovered in the porcine kidney cell line PK-15 [6] and is generally known to not be pathogenic [7]. In the 1990s, PCV2 was first discovered [8] and has been linked to a variety of clinical manifestations in pigs called Porcine Circovirus-Associated Disease (PCVAD). Subsequently, PCV3 was discovered in the US in 2016 [9,10], and has been the focus of much interest and research. Then, PCV4 was recently identified as a type in 2019 [11], and more information was needed to understand its potential impact.

In 2016, an emerging porcine viral pathogen known as PCV3 was discovered [10] to be associated with several clinical presentations in pigs, including PDNS, reproductive failure, and multi-systemic inflammation, which are similar to PCV2. Thereafter, many retrospective studies demonstrated that PCV3 has also been detected in North America, South America, Europe, and Asia [12,13,14,15,16,17], including China, indicating that this pathogen has been extensive in swine herds for a long time. However, there has only been one successful PCV3 isolation in cell culture that has been published to date [18], and isolating and propagating PCV3 in continuous cell lines was considered the primary barrier to studying its pathogenesis.

Like any other circovirus, PCV3 has a small genome of 2000 nucleotides, which encodes at least three open reading frames (ORFs) [9,10], ORF1 to ORF3, respectively. The ORF1 gene encodes the replication-associated protein (Rep), which makes up the most conserved region in the genome; ORF2 encodes the only viral capsid protein (Cap) that mainly induces the host immune response in circoviruses. The function of ORF3 is still unknown [10]. Increasing studies have shown that the Cap protein plays a major role in the antigenicity characteristics, and the two amino acid mutations (A24V and R27K) in the protein were employed as molecular markers for the subtype classification of PCV3 strains [19]. Through phylogenetic analysis, PCV3 isolates around the world could be divided into three subtypes (PCV3a, PCV3b, and PCV3c) according to the amino acid sequence of the Cap protein to date [20,21,22]. Therefore, investigating the amino acid sequence in this protein can improve our understanding of PCV3 evolution.

Although PCV3 has already been described in China, knowledge of available measures against this infectious disease is limited. For a better understanding of PCV3 epidemiological characteristics, we systematically investigated PCV3 prevalence in China and elucidated its genetic diversity and evolution. In the present study, 2707 serum samples of pigs were randomly collected from 17 provinces in China between September 2018 and March 2022, to detect the prevalence of PCV3 DNA. Furthermore, the ORF2 gene (for Cap) of 22 PCV3 strains that were analyzed in this study provided further evidence for the genetic divergence of PCV3 in China. Together, our findings will not only be important for understanding the prevalence and evolution of PCV3 in China but will also contribute to providing scientific data for developing effective strategies against the disease.

## 2. Materials and Methods

### 2.1. Sample Collection

**Figure 1:**
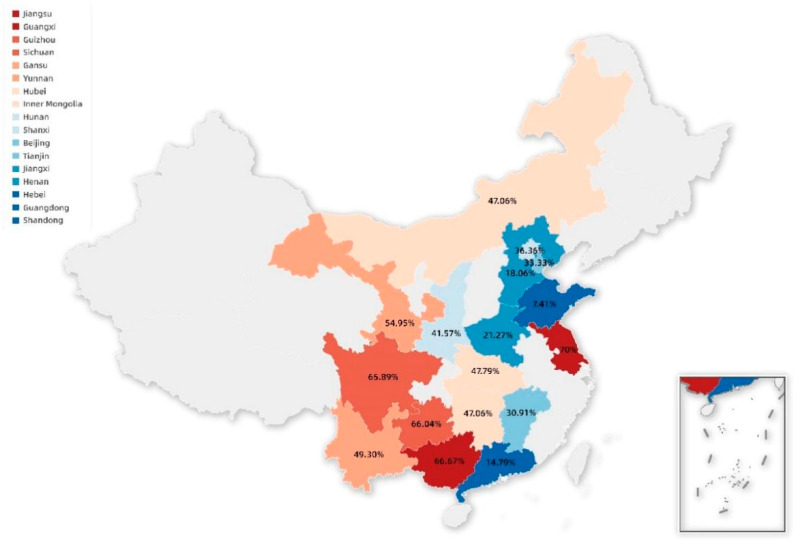
PCV3 strains’ geographical distribution by province in China. Color markers represent PCV3-positive provinces and the corresponding positive rate in this study, whereas light gray indicates that PCV3 detection has not been carried out in these provinces.

To understand the prevalence of PCV3 in China, between September 2018 and March 2022, 2707 serum samples of pigs were collected from 17 provinces in China, including Hebei, Guangdong, Henan, Shandong, Guangxi, Hubei, Sichuan, Inner Mongolia, Gansu, Shanxi, Yunnan, Jiangxi, Guizhou, Beijing, Tianjin, Hunan, and Jiangsu. These samples were collected from pigs with the following criteria. (1) Regional differences: more samples were collected in major pig-producing provinces of China such as Hebei, Guangdong, Henan, Shandong, and Guangxi provinces, and fewer samples were collected in other pig-producing provinces of China such as Tianjin, Beijing, and Jiangsu provinces, and so on. (2) Up to ten samples were collected from each age group on a farm, if possible. As a standard procedure according to the literature [23], our laboratory collected samples from each farm and stored them at −80 °C until used. In Figure 1 and Table 1, detailed information on the samples collected from different provinces is provided.

### 2.2. DNA Extraction and PCR

According to the manufacturer’s recommendations (Blood & Tissue Kit, TIANGEN, Beijing, China), the viral DNA was extracted and stored at −20 °C. Then PCR assays were performed to diagnose PCV3 with a pair of specific primers (5′-GTCGCCACCGGGGGTCAGATTTA-3′ and 5′-GCTATGCCAGAAGAAGACTATTCAT-3′) that amplify the capsid gene in a 25 µL final reaction volume. In brief, all the PCR reactions were carried out by the PCR system (Thermal), and the following components were used in the amplification reaction: 2 × Taq Master Mix (Vazyme) was 12.5 µL, each primer (10 µM) was 0.5 µL, DNA was 2 µL, and ddH_2_O was 9.5 µL to a final volume. The PCR was conducted by using the following conditions: 94 °C for 5 min, followed by 30 cycles of 94 °C for 30 s, 56 °C for 30 s, and 72 °C for 30 s, and then a final extension of 7 min at 72 °C. The amplified products were analyzed, and positive products were purified and sequenced.

### 2.3. Gene Sequencing

To analyze the genetic divergence of PCV3 in China, 22 samples with strongly positive PCV3 from different regions were used to amplify the ORF2 gene with a pair of specific primers (5′-CATGCGAGGGCGTTTACCTG-3′and 5′-TCCCTACAGACCTCCGTGGATC-3′). The PCR reactions (50 µL) were performed under the following conditions: 25 µL of 2 × KOD One^TM^ PCR Master Mix (TOYOBO), 1.0 µL of each primer, 2.0 µL of DNA template, and 21 µL of ddH_2_O. The conditions were 95 °C for 3 min, 35 cycles at 98 °C for 10 s, 58 °C for 5 s, and 68 °C for 5 s, followed by final elongation at 68 °C for 10 min. The PCR products were purified and sequenced (Tsingke Biotechnology, Beijing, China).

### 2.4. Bioinformatics Analyses

In order to better understand the PCV3 strains’ genetic characteristics, the PCV3 sequences obtained in our study and the 24 PCV3 reference strains, which originated from different countries and were downloaded from the NCBI nucleotide database, were used and aligned by using Lasergene software with the Clustal W program for homology analysis. A phylogenetic tree of the ORF2 gene was generated by using the neighbor-joining method in MEGA11 software [24], and an ML tree by using over 10,000 bootstrap iterations to represent the evolutionary history of the taxa analyzed [25]. Gene sequence and amino acid sequence alignments of the ORF2 gene were analyzed by using BioAider V1.423 [26].

## 3. Results

### 3.1. The Prevalence of PCV3 in China

To better understand the epidemiology of PCV3 in China, 2707 serum samples of pigs in 17 provinces of China from September 2018 to March 2022 were evaluated by PCR assays. As shown in Table 1, PCV3 infection was prevalent in the overall population with 31.07% (841/2707) and 100.0% (17/17) at the sample and province levels, respectively. However, the positivity rate of PCV3 in different provinces was different and varied from 7.41 to 70.0%, with the relatively higher prevalence of PCV3 observed in the Jiangsu (70.0%, 7/10), Guangxi (66.67%, 148/222), Guizhou (66.04%, 35/53), and Sichuan (65.89%, 85/129) provinces. Meanwhile, the prevalence of PCV3 was 0.0%, 1.21%, 7.25%, 38.8%, and 50.0% in 2018, 2019, 2020, 2021, and 2022, respectively (Table S1), indicating the prevalence of PCV3 increased year by year in our study.

### 3.2. Bioinformatics Analysis

**Figure 2:**
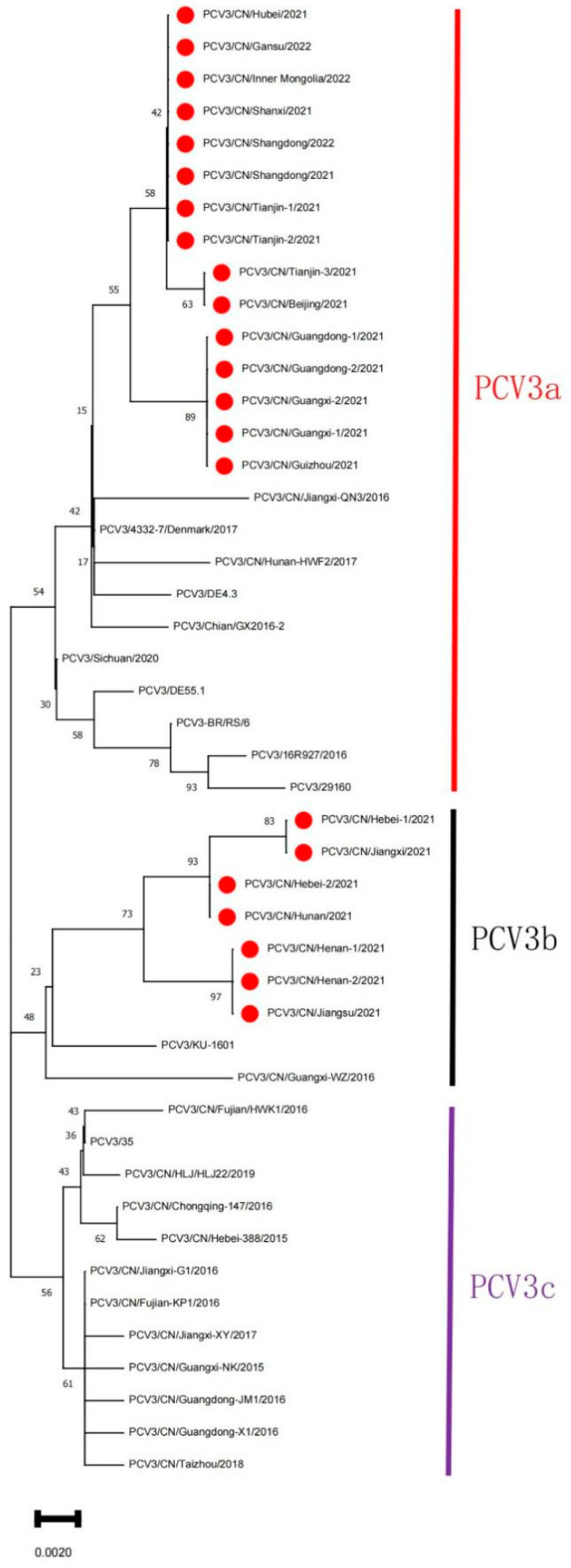
Phylogenetic analysis of the ORF2 gene of PCV3 strains. The PCV3 sequences were aligned by using Lasergene software with the Clustal W program for homology analysis. The evolutionary history was inferred by using the neighbor-joining method in MEGA11 software [24], and an ML tree by using over 10,000 bootstrap iterations to represent the evolutionary history of the taxa analyzed [25]. This analysis involved 46 nucleotide sequences, including the 22 PCV3 strains identified in our study and 24 PCV3 reference strains downloaded from the NCBI. Red circles represent PCV3 strains obtained in this study.

To investigate the genetic characteristics of the PCV3 strains, the sequences obtained in our study and 24 PCV3 reference strains downloaded from the NCBI nucleotide database were analyzed. Phylogenetic analysis based on the ORF2 sequences revealed that three subtypes could be distinguished (PCV3a, PCV3b, and PCV3c) among the PCV3 strains available (Figure 2), and all isolates in the current study could be grouped into two separate subclades, with 15 strains belonging to clade 3a and 7 strains belonging to clade 3b. Meanwhile, the gene sequence and amino acid sequence alignments indicated that the gene sequences identified in this study displayed 97.46~99.8% nucleotide (nt) and 97.06~100% amino acid (aa) sequence similarity (Table S2) with other PCV3 reference strains, respectively.

## 4. Discussion

PCV3, as a newly emerging porcine viral pathogen, has been proven to cause several clinical symptoms in pigs similar to PCV2, including multisystem failure syndrome, dermatitis of weaned piglets, porcine dermatitis nephropathy syndrome (PDNS) [9], reproductive failure [27], and respiratory disorders [28,29,30], etc. Since the first identification of PCV3 in the United States, growing evidence indicates PCV3 has also been detected in Asia, North America, South America, and Europe [12,13,14,15,16,17]. In view of the huge losses caused by PCV3 to the agriculture industry, like PCV2, it is urgent to carry out an epidemiological investigation into PCV3 to understand its infection rate and epidemic areas.

PCV3 was first identified in aborted fetuses of sows with dermatitis and nephrotic syndrome in the United States, and 34 out of 271 clinical samples associated with reproductive disorders were positive, with a positive detection rate of 12.55% [9]. Previous studies in China have reported 77 positive samples from 222 clinical samples collected from 35 pig farms in 11 regions, including Chongqing, Liaoning, and Jiangxi, with a positive rate of 34.7% (77/222) and a co-infection rate of 15.8% (35/222) for PCV2 and PCV3 [14]. The epidemiological investigation of PCV3 in southern China showed that a prevalence of PCV3 was 26.7% (76/285), and the prevalence rate of aborted fetuses was the highest through PCR assays [31]. Another study showed that the prevalence of PCV3 in southern China was 21.9%, 27.8%, and 31.1% in 2015, 2016, and 2017, respectively [19]. Compared with these studies, our results came from an increased number of samples and a wider geographical distribution. These serum samples were collected in the last five years, representing the latest prevalence trend of PCV3, and the number has reached more than 2700, involving 17 provinces across the country with a wide geographical representation. In this study, an average PCV3 prevalence of 31.07% (841/2707) was observed in all of the provinces, which is consistent with recent studies and reflects a high prevalence of PCV3, indicating that PCV3 is highly prevalent among Chinese pigs. However, the positivity rate of PCV3 was different in provinces, ranging from 7.41 to 70.0%, which might be due to the limited sample size. In addition, we also found that the prevalence of PCV3 increased year by year, and the positive rate in 2022 was significantly higher than in other years (Table S1). We hypothesized that it might be related to the outbreak and prevalence of African swine fever (ASF), which was introduced into China after September 2018, and which has had a serious impact on the domestic pig industry.

Although the number of available sequences in PCV3 is increasing, the analysis results are very similar to those reported previously. PCV3 strains share high sequence homology based on the sequences of available strains from different countries [16,31,32,33,34], and are highly homologous to bat circovirus [20,21]. A rapid increase and expansion of the PCV3 population from late 2013 to early 2014 was revealed by the skyline plot. Meanwhile, PCV3a and PCV3b were formed during this period [20]. Multiple PCV3 strain subtypes around the world have been reported to date, but the genotype of PCV3 is still controversial because of the inconsistent genotyping method used. In some studies, PCV3 could be divided into three major clades (PCV3a, PCV3b, and PCV3c) based on the full-length genomic DNA sequence or the amino acid mutations in the Cap protein [19,31,35,36]. In addition, a group from China found that PCV3 is divided into two genotypes, PCV3a and PCV3b, and a flexible IM clade and two stable subclades were identified within the PCV3a clade [20,21]. Overall, these results indicate the increasing genetic diversity of PCV3 in the swine population. In this study, phylogenetic analysis based on the nucleotide sequence of ORF2 was performed, and PCV3 strains prevalent globally were divided into three clusters, namely PCV3a, PCV3b, and PCV3c (Figure 2), including Chinese strains and strains from Asia, the Americas, and Europe, suggesting that PCV3 strains are dispersed globally. In addition, all isolates in the current study could be grouped into two separate subclades, with 15 strains belonging to clade 3a and 7 strains belonging to clade 3b, indicating that PCV3a and PCV3b were the predominant subtypes in the regions of China studied, which will guide future efforts to develop measures for preventing and controlling the disease. Future experiments should examine the pathogenic characteristics of the two phylogenetic clades as well as the factors that led to the rapid molecular evolution of PCV3.

## 5. Conclusions

In general, we detected the prevalence of PCV3 on pig farms with prolonged histories in different provinces of China and further analyzed the genetic divergence of the ORF2 gene of PCV3 strains in the present study. Overall, our study provides important insights for understanding the prevalence and evolution of PCV3 in China and will contribute to future efforts to develop measures for preventing and controlling the disease.

## Figures and Tables

**Table 1:** Prevalence of PCV3 in serum samples from different provinces of China by PCR.

Scheme	Sample Numbers	Positive Samples	Positive Rate (%)
Hebei	587	106	18.06
Guangdong	399	59	14.79
Henan	362	77	21.27
Shandong	270	20	7.41
Guangxi	222	148	66.67
Hubei	136	65	47.79
Sichuan	129	85	65.89
Inner Mongolia	119	56	47.06
Gansu	91	50	54.95
Shanxi	89	37	41.57
Yunnan	71	35	49.30
Jiangxi	55	17	30.91
Guizhou	53	35	66.04
Beijing	44	16	36.36
Tianjin	36	12	33.33
Hunan	34	16	47.06
Jiangsu	10	7	70.00
Total	2707	841	31.07

## Data Availability

The data that support the findings of this study are available on request from the corresponding author.

## Supplementary Materials

The following supporting information can be downloaded at: https://www.mdpi.com/article/10.3390/ani12121588/s1, Table S1: Prevalence of PCV3 in serum samples from different years of China by PCR; Table S2: Similarities of nucleotides (nt) and amino acids (aa) of the ORF2 genome (Cap) of PCV-3 sequences (%).
