# Supplementary material for: Prevalence and Evolution Analysis of Porcine Circovirus 3 in China from 2018 to 2022
Source: Animals (Basel). 2022 Jun 20;12(12):1588. doi: 10.3390/ani12121588 (PMC9219504; doi:10.3390/ani12121588)
Supplement: Supplementary file 1 [file animals-12-01588-s001.zip › animals-1712288-supplementary/Table S1.pdf]

Table S1. Prevalence of PCV3 in serum samples from different years of China by PCR

Collection date	Specimen numbers	Positive samples	Positive rate (%)
**2018**	71	0	0.00
**2019**	247	3	1.21
**2020**	345	25	7.25
**2021**	1880	731	38.88
**2022**	164	82	50.0
**Total**	2707	841	31.07
