# Supplementary material for: Prevalence and Evolution Analysis of Porcine Circovirus 3 in China from 2018 to 2022
Source: Animals (Basel). 2022 Jun 20;12(12):1588. doi: 10.3390/ani12121588 (PMC9219504; doi:10.3390/ani12121588)
Supplement: Supplementary file 1 [file animals-12-01588-s001.zip › animals-1712288-supplementary/Table S2.pdf]

Table S2. Similarities of nucleotides (nt) and amino acids (aa) of the ORF2 genome (Cap) of PCV-3 sequences (%)

Representative PCV-3 Strains				PCV-3 Strains Obtained in This Study													
Sub-clade	GenBank accession no.	Strain name	Collection date	CN/Tianjin-1/2021		CN/Tianjin-2/2021		CN/Tianjin-3/2021		CN/Guangdong/2021		CN/Hebei-1/2021		CN/Hebei-2/2021		CN/Henan/2022	
				nt	aa	nt	aa	nt	aa	nt	aa	nt	aa	nt	aa	nt	aa
	MF805724.1	4332-7/Denmark/2017	2017	99.8	98.27	99.8	98.27	99.61	97.69	99.61	97.69	98.24	95.95	98.63	96.53	98.63	96.53
	MF155642.1	Chian/GX2016-2	2016	99.41	97.11	99.41	97.11	99.22	96.53	99.22	96.53	97.85	94.8	98.24	95.38	98.24	95.38
	MF079253.1	BR/RS/6	2016	99.02	96.53	99.02	96.53	98.83	95.95	98.83	95.95	98.04	94.8	98.24	94.8	98.24	94.8
	MF063071.1	16R927/2016	2016	98.63	95.38	98.63	95.38	98.43	94.8	98.43	94.8	97.65	93.64	97.85	93.64	97.85	93.64
3a	KT869077.1	29160	2015	98.43	94.8	98.43	94.8	98.24	94.22	98.24	94.22	97.46	93.06	97.65	93.06	97.65	93.06
	MF589132.1	CN/Jiangxi-QN3/2016	2016	99.02	97.11	99.02	97.11	98.83	96.53	98.83	96.53	97.46	94.8	97.85	95.38	97.85	95.38
	MF589127.1	CN/Hunan-HWF2/2017	2017	99.22	96.53	99.22	96.53	99.02	95.95	99.02	95.95	97.65	94.22	98.04	94.8	98.04	94.8
	MG014363.1	DE4.3	2015	99.41	97.69	99.41	97.69	99.22	97.11	99.22	97.11	97.85	95.38	98.24	95.95	98.24	95.95
	MG014376.1	DE55.1	2015	99.22	97.11	99.22	97.11	99.02	96.53	99.02	96.53	98.04	94.8	98.43	95.38	98.43	95.38
	MZ449244.1	Sichuan/2020	2020	99.61	98.27	99.61	98.27	99.41	97.69	99.41	97.69	98.43	95.95	98.83	96.53	98.83	96.53
3b	MF589123.1	CN/Guangxi-WZ/2016	2016	98.24	94.8	98.24	94.8	98.43	95.38	98.04	94.22	98.24	94.8	98.63	95.38	98.24	95.38
	KY996337.1	KU-1601	2016	98.83	95.38	98.83	95.38	99.02	95.95	98.63	94.8	98.43	96.53	98.83	97.11	98.83	95.95
	MF589131.1	CN/Jiangxi-G1/2016	2016	99.22	97.11	99.22	97.11	99.02	96.53	99.02	96.53	98.43	95.95	98.83	96.53	98.43	95.38
	MF589134.1	CN/Jiangxi-XY/2017	2017	99.02	97.11	99.02	97.11	98.83	96.53	98.83	96.53	98.24	95.95	98.63	96.53	98.24	95.38
	MF589122.1	CN/Guangxi-NK/2015	2015	99.02	96.53	99.02	96.53	98.83	95.95	98.83	95.95	98.24	95.38	98.63	95.95	98.24	94.8
	MF589115.1	CN/Guangdong-JM1/2016	2016	99.02	96.53	99.02	96.53	98.83	95.95	98.83	95.95	98.24	95.38	98.63	95.95	98.24	94.8
	MF589118.1	CN/Guangdong-X1/2016	2016	99.02	97.11	99.02	97.11	98.83	96.53	98.83	96.53	98.24	95.95	98.63	96.53	98.24	95.38
3c	MF589111.1	CN/Fujian-KP1/2016	2016	99.22	97.11	99.22	97.11	99.02	96.53	99.02	96.53	98.43	95.95	98.83	96.53	98.43	95.38
	MF589109.1	CN/Fujian/HWK1/2016	2016	98.83	96.53	98.83	96.53	98.63	95.95	98.63	95.95	98.04	95.38	98.43	95.95	98.04	94.8
	MN788132.1	CN/HLJ/HLJ22/2019	2019	99.02	97.11	99.02	97.11	98.83	96.53	98.83	96.53	98.24	95.95	98.63	96.53	98.24	95.38
	MW167067.1	35	2020	99.22	97.11	99.22	97.11	99.02	96.53	99.02	96.53	98.43	95.95	98.83	96.53	98.43	95.38
	MK580466.1	CN/Taizhou/2018	2018	99.02	96.53	99.02	96.53	98.83	95.95	98.83	95.95	98.24	95.38	98.63	95.95	98.24	94.8
	KY075990.1	Chongqing-147/2016	2016	99.02	96.53	99.02	96.53	99.22	97.11	98.83	95.95	98.63	96.53	99.02	97.11	98.63	95.95
	MF139083.1	CN/Hebei-388/2015	2015	98.83	95.95	98.83	95.95	99.02	96.53	98.63	95.38	98.43	95.95	98.83	96.53	98.43	95.38
